# A Competency-Guided Approach to Optimizing a Physician-Scientist Curriculum

**DOI:** 10.1007/s40670-022-01525-w

**Published:** 2022-03-07

**Authors:** Lourdes Estrada, Megan A. Williams, Christopher S. Williams

**Affiliations:** 1 Department of Biochemistry, Vanderbilt University School of Medicine, Nashville, TN 37240 USA; 2 Medical Scientist Training Program, Office of Health Science Education, Room 222 Eskind Biomedical Library and Learning Center, 2209 Garland Avenue, Nashville, TN 37240 USA; 3 Department of Pharmacology, Vanderbilt University School of Medicine, Nashville, TN USA; 4 Department of Medicine, Div. of Gastroenterology, Vanderbilt University Medical Center, 2215 Garland Avenue, 1030C MRB IV, Nashville, TN 37232 USA

## Abstract

Physician-scientists are uniquely positioned to achieve significant biomedical advances to improve the human condition. Their clinical and scientific training allows them to bridge fields and contribute to cutting-edge, clinically relevant research. The need for a highly skilled physician-scientist workforce has never been more acute. We propose a competency-guided program design (CGPD) framework that focuses on core skills to enhance the physician-scientist training curriculum. In partnership with clinical and graduate curricula, the CGPD framework can be employed as a tool to meaningfully integrate physician-scientist training, address barriers to attract and sustain the physician-scientist workforce, and avoid overprogramming that detracts from a solid foundation of clinical and graduate research training.

## Background

As clinical and research disciplines have become more specialized and sophisticated, the training of physician-scientists has evolved, better-preparing trainees for increasingly diverse careers. The NIH Physician-Scientist Workforce Working Group Report released in 2014 and numerous following publications have described and documented remaining challenges facing physician-scientist [1–3]. These challenges include funding research programs, mentorship, and personal development and wellness.

Physician-scientist curricula are changing in response to documented challenges to attract and sustain the workforce as well as benchmarks established by competing programs, student-led initiatives, institutional requirements, Liaison Committee on Medical Education (LCME) clinical training requirements, training program evaluation, and the blending of updated clinical and graduate training activities. While these adaptations have led to numerous innovations, they can result in diminished curriculum integration, overprogramming, redundant or irrelevant training activities, a loss of training focus, and increased administrative burden. While innovations to physician-scientist training activities help optimize skill development, increase recruitment, and maximize retention, curricular design and refinement should be guided by systematic approaches based on overall program training objectives [2].

## Competency-Based Education

Competency-based education (CBE) de-emphasizes time-based training and provides a learner the opportunity to advance at their own pace based on their ability to master particular skills [4, 5]. Competencies include explicit, measurable, transferable learning objectives that empower trainees to understand what is expected for mastery and advancement. In addition, an essential component of CBE is timely, personalized support for each student based on individual learning needs.

In 1999, the Accreditation Council for Graduate Medical Education (ACGME) and the American Board of Medical Specialties (ABMS) endorsed six core clinical competency domains: Patient Care, Medical Knowledge, Professionalism, Interpersonal and Communication Skills, Practice-Based Learning and Improvement, and Systems-Based Practice. The Vanderbilt University School of Medicine (VUSM) implemented the ACGME competency-based assessment in 2013 at the undergraduate medical education (UME) level, including evidence-based curricula [6, 7]. Competency-based education carefully aligns assessment with the acquisition of competencies expected of graduating physicians.

Graduate training programs have been slower than medical schools to implement CBE, but change is imminent. [8, 9] The NIH recently modified the requirements for graduate training programs to “identify training needs and objectives (i.e., specific, measurable outcomes the program intends to achieve), and develop plans to implement evidence-based training activities.” The new funding opportunity announcement (FOA) also challenges funded programs “to provide evidence of accomplishing the training objectives.” The initial step in accomplishing these overarching goals is to define these training objectives in terms of attained competencies.

**Fig. 1:**
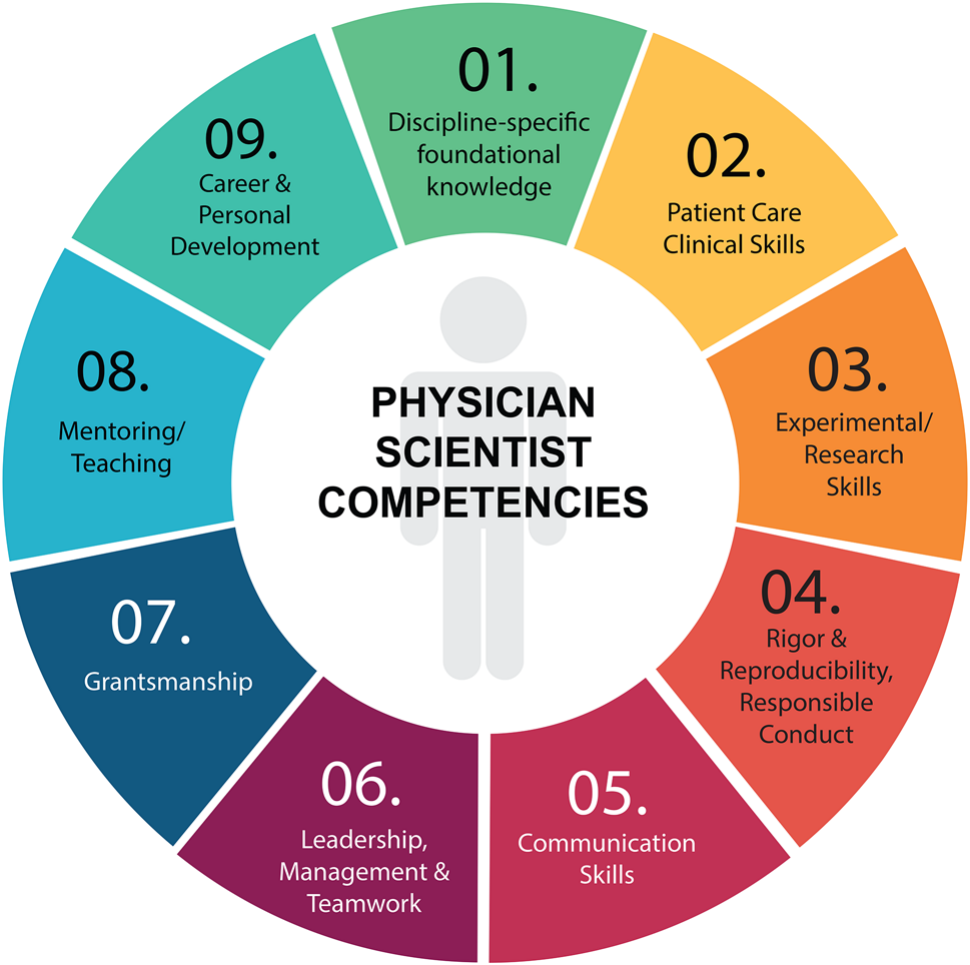
Proposed nine core competencies for physician-scientists

Physician-scientists address critical medical care and human health issues by integrating clinical practice and research activities into their careers. Consequently, their training curriculum must comprise a strong core education in clinical medicine and intensive training in scientific inquiry. Based on our experience, we developed a Competency-Guided Program Design (CGPD) framework to align our curriculum with nine core competencies (Fig. 1) that prepare physician-scientists to translate clinically driven research activities into medical advances.

## Physician-Scientist Competencies

**Fig. 2:**
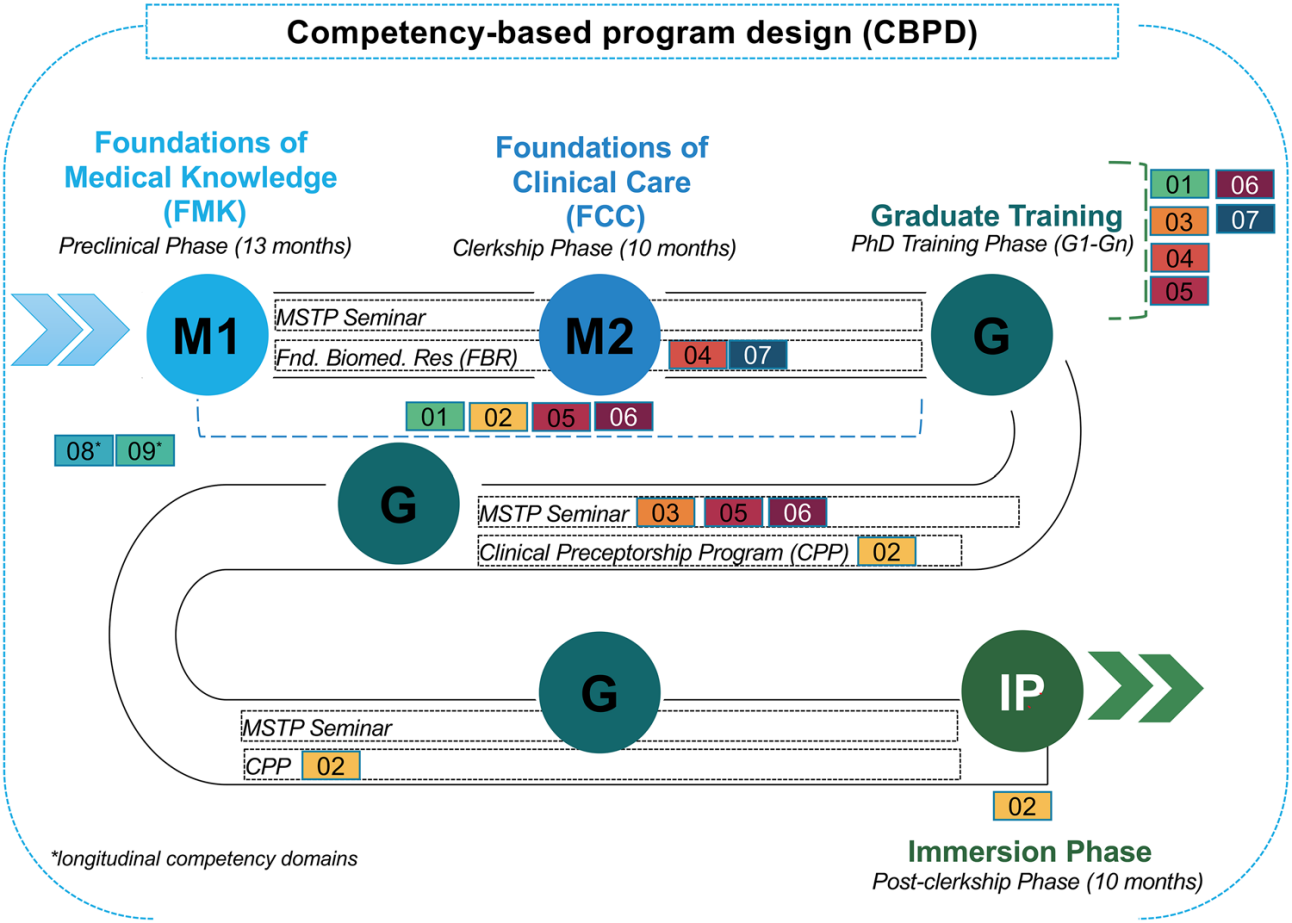
Mapping of the competency domains on the Vanderbilt MSTP Curriculum. The Vanderbilt MSTP integrates rigorous clinical and doctoral graduate training in a 7-year training program. In the first two years (medical school years 1 and 2, M1 and M2), our trainees follow the VUSM C2.0 curriculum with all MD pathway students. During these two years, training is focused on the depicted core competencies (numbers correlating to core competency domain in Fig. 1). After a dedicated time for United States Medical Licensing Examination (USLME) Step 1/2 study time, our trainees enter graduate school (G1–Gn) in one of the 16 doctoral programs affiliated with our MSTP. After completion of their doctoral graduate program requirements, including thesis defense, our students return to one final year of medical school training (immersion phase, IP). Two competency domains are highlighted as longitudinal as these are integrated throughout training for physician-scientists: mentoring/teaching (08) and career and personal development (09). The map also highlights several important MSTP-specific courses that have been designed using the CGPD framework: (a) MSTP Seminar Series, (b) Foundations of Biomedical Research, and (c) Clinical Preceptorship Program (CPP). CPP provides MSTP students with exposure to clinical medicine during the period of research training to practice clinical skills developed during the clerkship M2 year, explore subspecialties of interest to help determine a target residency track, and identify potential clinical mentorship in the area of their future clinical training [11]

The Vanderbilt University Medical Scientist Training Program (MSTP) integrates the clinical and graduate educational programs by providing a framework for trainees to develop and practice competencies deemed critical for successful physician-scientist careers. The map in Fig. 2 depicts our training program in which students complete the requirements to receive both the M.D. and Ph.D. degrees. MSTP students typically complete the Foundations of Medical Knowledge (medical school year 1, M1) and Foundations of Clinical Care (medical school year 2, M2) phases, engage in graduate studies (graduate years, G), and then return for a single remaining medical school year (M4), the Immersion Phase. Although each training phase has a primary core competency focus, there is intentional overlap embedded as the application of multiple competencies is uniquely viewed through the physician and scientist lenses and is implemented longitudinally throughout training (i.e., mentoring/teaching and career/personal development).

The nine core competency domains (CD, Fig. 1**)** were derived from published desired competencies from graduate and medical education. [9, 10] Importantly, trainees provide longitudinal feedback on competency domains and reinforce their use when planning programmatic activities, including student-led initiatives. Within each of these nine domains, we address multiple skill sets such as oral and written communication, translation of clinical questions to research, management of challenges and conflict resolution, teamwork and group dynamics, wellness, self-knowledge, the balance of clinical and research responsibilities, best mentoring practices, scientific promotion, advocacy, and policy. Although some of these domains may already be addressed in both medical (e.g., patient care) and graduate (e.g., communication skills) curricula, including these domains in the physician-scientist curricula reinforces their importance and allows for deeper integration of these skills across various training experiences and learning environments.

## Advantages of Competency-Guided Program Design

**Fig. 3:**
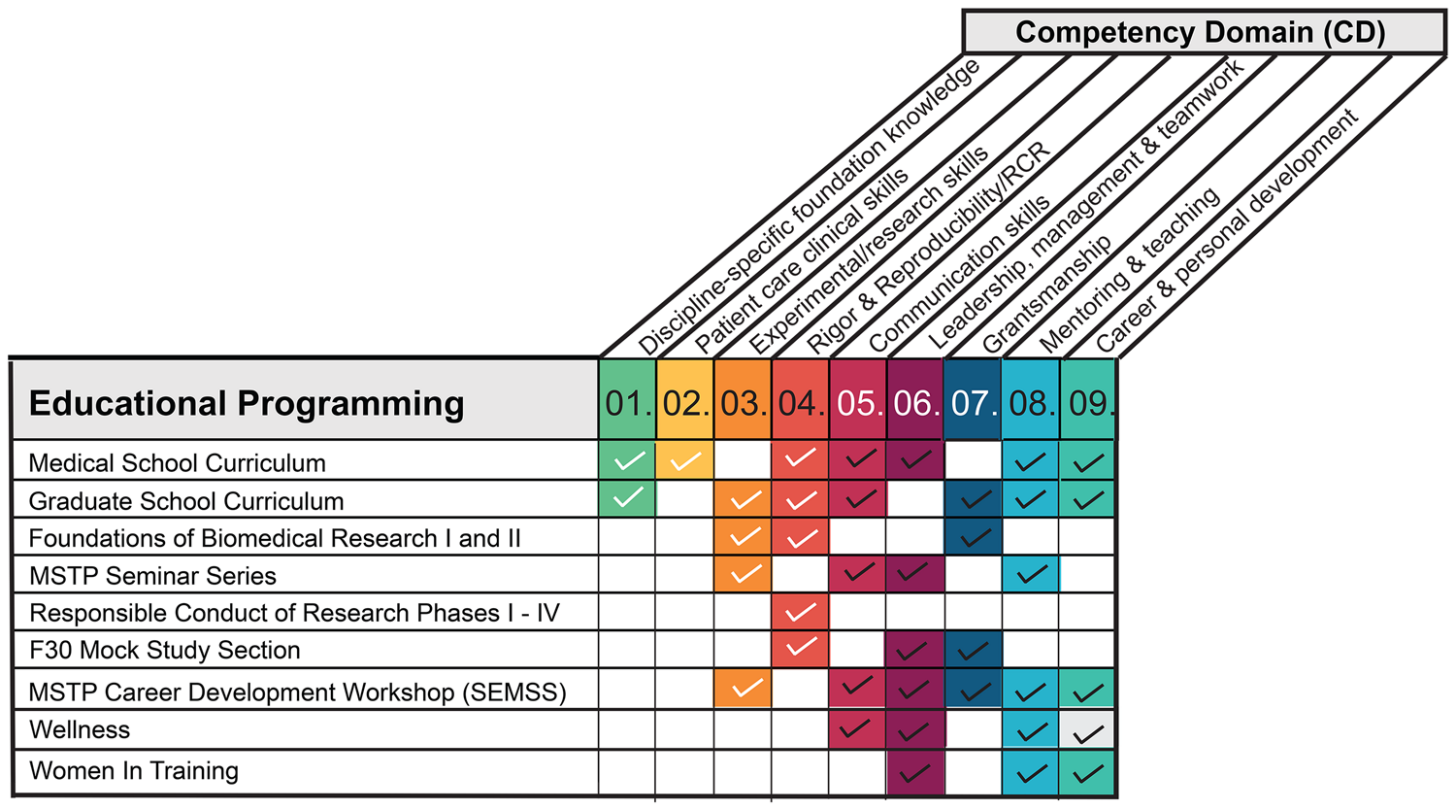
Example matrix of core competencies mapped by educational programming in the Vanderbilt MSTP. Color codes of competency domains (CD) are CD01: discipline-specific foundational knowledge; CD02: patient care clinical skills; CD03: experimental/research skills; CD04: rigor and reproducibility and responsible conduct; CD05: communication skills; CD06: leadership and management; CD07: grantsmanship; CD08: mentoring/teaching; and CD09: career and personal development

There are several advantages to using a competency-guided program design (CGPD) approach. First, a CGPD approach facilitates the intentional configuration of learning activities to maximize trainee competency acquisition. CGPD provides a framework to deliberately map programming activities to specific competency domains to ensure alignment with desired training objectives (Fig. 3). Second, the CGPD approach facilitates quality improvement by exposing gaps in curriculum design that result in insufficient training in the desired competency domain. Conversely, it aids in identifying programming misaligned with essential competencies necessary to train physician-scientists and allows the retiring of these activities. CGPD provides a tool to avoid overprogramming, a natural outcome given the heavy emphasis on innovation and enhancement during program review processes. Third, mapping proposed new activities to specific competencies serves as a litmus test to determine if new programming is advantageous and justified. Fourth, using the CGPD framework provides valuable information to trainees as they seek to develop their physician-scientist careers. Fifth, CGPD can aid program evaluation, a mandatory component of NIH T-32 supported training programs, by clearly articulating the desired program outcomes (short-, mid-, and long-term) aligned with the necessary resources (inputs) and educational activities. Finally, CGPD aids in identifying opportunities for greater integration between clinical and graduate training curricula, thus reinforcing and translating learned skills in different professional environments.

Overall, by addressing the issues listed above, the CBPD framework allows programs to directly tackle challenges and barriers associated with physician-scientist careers [1–3]. For example, programs can be intentional in addressing funding challenges by establishing a grantsmanship curriculum specifically designed to develop skills critical to securing research funding as trainees’ careers progress. Programs can also facilitate mentor development by implementing formalized mentoring programs, including “mentoring the mentor” training. Finally, CGPD can raise the importance of personal development and wellness skills that will facilitate work-life balance and minimize burnout.

The Vanderbilt University MSTP uses CGPD to fine-tune our curriculum. After defining the competency domains, we mapped all core curriculum and programmatic activities to each domain (Fig. 3) and then adjusted programming as indicated. We provide changes made to the Vanderbilt MSTP curriculum to illustrate the CGPD approach.

Applying CGPD to the weekly MSTP Seminar Series, the flagship course of our educational program, which extends throughout the medical and graduate phases of training, we mapped the series learning objectives to the core competencies for physician-scientists (Fig. 3). The series is a weekly, student-led, literature-based course consisting of journal club, research in progress, and clinical-based presentations. The objectives of the series are to (a) foster development of critical-thinking skills by appraisal of contemporary scientific literature; (b) enhance scientific creativity through discussion of experimental approaches and techniques; (c) develop oral communication skills; (d) afford weekly opportunities for interaction between MSTP trainees and the Vanderbilt physician-scientist community; and (e) provide practical leadership and peer-peer mentoring experiences through organizing and running the series. Mapping these learning objectives to the core competency domains facilitated refining the series format to ensure programming achieved the series objectives. For example, first-year MSTP student journal club talks were modified to enhance mentor–mentee relationships (mentoring/teaching), appraising a scientific paper (discipline-specific foundational knowledge), and developing presentation skills (communication skills).

Our competency-based curriculum map identified a gap in our Career and Personal Development domain, which led to deliberate efforts to implement wellness and women-in-training programming to address the mental health and gender equity needs specific to the MSTP community and physician-scientist training. To close the identified gap, we developed faculty-guided, student-led committees tasked with enhancing the wellness of the student body through programming that decreases mental health stigma, provides resources, educates trainees about the challenges facing physician-scientists, and provides social interactions to foster near-peer and peer-peer mentoring relationships. These highly rated programs are now in their 3rd year.

The CGPD framework with curriculum mapping has also identified overprogramming. For example, with the implementation of the MSTP Mock Study Section (a student-led, faculty moderated, fellowship review committee modeled after NIH review panels) and increasing grantsmanship training through formal coursework and seminars in graduate school, we retired our annual MSTP Grant Writing Workshop.

Finally, CGPD can be a guiding mechanism in evaluating training programs. The NIH MSTP T32 funding mechanism now requires formal program evaluation using evidence-based approaches. The NIH defines program evaluation as a “systematic assessment of the operations and/or outcomes of a program, compared to a set of explicit or implicit stands, as a means of contributing to the improvement of the program.” [12, 13] The NIH also requires every funded M.D.-Ph.D. training program to “describe an evaluation or assessment process to determine whether the overall program is effective in meeting its training mission and objectives.”[14] Formal program evaluation models offer numerous benefits by (1) providing a clear definition of the program’s scope; (2) improving planning, management, and quality improvement; and (3) augmenting intentionality and purpose [15, 16]. The CGPD offers a framework to define an evaluation model based on program components (e.g., resources, activities, outputs) and desired outcomes mapped to short, intermediate, and long-term competencies. [17].

The CGPD is a conceptual overlay structure for any program that trains physician-scientists and implementation as such does not require significant infrastructure. The implementation would usually consist of incorporation into existing programmatic structure and workflows as curricular changes are being considered. Overall, the implementation of CGPD optimizes and streamlines program development, thus focusing activities on maximizing the physician-scientist training experience.

## Future Directions

### Refining Competencies

The competency domains designed to guide medical student training were first adopted in 1999 by the ACGME focus on the physician’s role and their ability to provide high-quality patient care. [11] Since their inception, the ACGME core competencies have continued to evolve, and other frameworks have been developed, such as the Entrustable Professional Activities (EPA). [18] The proposed nine core competencies for physician-scientists (Fig. 1) should also evolve to ensure that they continue to include all of the essential skills for success in the physician-scientist career path. Therefore, we believe that a robust national conversation regarding physician-scientists core competencies rooted in desired outcomes should commence balancing the efficiency of training with skill development throughout the continuum of physician-scientist education (from trainees to faculty).

### Assessment

As mentioned above, defining and adopting core competencies is an initial step in CGPD. To assess trainee competency attainment, the development of validated milestone-based instruments is an essential next step in the competency-based education of physician-scientists. Milestones are developmental behavioral descriptors of the learning trajectory within the core competencies that align expectations between the learner and the faculty. Currently, milestones are utilized primarily in graduate medical education (GME) to assess the development of residents and, to a lesser extent, in undergraduate medical education (UME) [7, 19, 20]. Biomedical graduate training programs lack validated milestone-based assessment methods and have only begun to explore the milestone framework [9]. CGPD provides an opportunity for graduate doctoral programs to respond proactively to the NIH’s call for the development and implementation of “effective, evidence-based approaches to biomedical graduate training.” [19] While the individual development plan (IDP) is one tool that has been successfully implemented to help support, plan, and track trainees’ career development and learning opportunities and to facilitate communication between mentees and mentors, [21] there is an opportunity to complement the IDP with milestone-based assessment. However, these methods differ in their primary approach. While the IDP is primarily used by mentees and mentors to plan and communicate progress, milestone-based assessments require multiple observers and learning settings (e.g., classroom, laboratory, department). In addition, they are designed to monitor and improve educational outcomes at the individual learner level by modifying the training program structure. Thus, the natural companion to CGPD is building in assessment tools to track trainee progress towards competency attainment to a level beyond the stage of proficiency.

## Conclusions

Why should you consider the CGPD approach described here to refine your program design? We have observed that the CGPD framework facilitates optimization of physician-scientist education anchored on core competencies that build upon a solid integrated foundation of clinical and graduate research training. It does this by (1) ensuring that activities align with desired outcomes, (2) exposing gaps in training, (3) avoiding overprogramming, (4) informing new programming, (5) guiding program evaluation, and (6) identifying opportunities for greater integration. Notably, the trainees also benefit from CGPD because the program’s expectations (and career outcomes) are clearly articulated, therefore allowing trainees to assess their progress and design personalized learning goals. Although this manuscript is focused on the application of the CGPD to dual degree (M.D./Ph.D.) programs, we posit that it can be applied to other graduate programs, including master’s level degree programs (e.g., master’s in clinical investigation, master’s in biomedical informatics, master’s in public health) and late bloomer physician-scientist programs (e.g., research in residency).

To conclude, we hope that the CGPD provides a springboard for critical national discussions on the core competencies that all trainees need to attain to become successful in their future careers as physician-scientists, effective assessment strategies to track our trainees’ progress, and build evaluation models that lead to intentional and impactful quality improvement efforts.
